# Negative Association between Acrylamide Exposure and Metabolic Syndrome Markers in Adult Population

**DOI:** 10.3390/ijerph182211949

**Published:** 2021-11-14

**Authors:** Chun-Chi Hung, Yung-Wen Cheng, Wei-Liang Chen, Wen-Hui Fang

**Affiliations:** 1 Department of Orthopaedic Surgery, Tri-Service General Hospital, Taipei 114, Taiwan; raffi10110126@gmail.com; 2 School of Medicine, National Defense Medical Center, Taipei 114, Taiwan; cyw0451@gmail.com; 3 Division of Family Medicine, Department of Family and Community Medicine, Tri-Service General Hospital, Taipei 114, Taiwan; 4 Division of Geriatric Medicine, Department of Family and Community Medicine, Tri-Service General Hospital, Taipei 114, Taiwan; 5 Department of Biochemistry, National Defense Medical Center, Taipei 114, Taiwan

**Keywords:** acrylamide, glycidamide, metabolic syndrome, NHANES

## Abstract

Metabolic syndrome encompasses multiple conditions that increase the risk of cardiovascular disease, and exposure to environmental chemicals can cause metabolic syndrome. This cross-sectional study analyzed data from the US National Health and Nutrition Examination Survey (2003–2006) on 4318 adult participants to assess the association between acrylamide (AA) exposure and metabolic syndrome. Concentrations of hemoglobin-adducted AA (HbAA) and hemoglobin-adducted glycidamide (HbGA) were evaluated. Metabolic syndrome markers related to HbAA and HbGA and the effect of exposure to AA and GA on the prevalence of metabolic syndrome were studied by ANOVA and multivariate logistic regression analyses, respectively. HbAA concentration inversely correlated with the number of metabolic syndrome markers (*p* < 0.05). An increased HbAA concentration was noted with reduced high triglyceride and low high-density lipoprotein cholesterol levels in the adjusted model (*p* < 0.05). High fasting plasma glucose level significantly correlated with HbGA concentration in the adjusted model. In conclusion, AA exposure alters metabolic syndrome markers in adults. Additional clinical and animal studies will clarify the role of AA exposure at different stages in the progression of metabolic syndrome-related diseases.

## 1. Introduction

Acrylamide (AA) is used to synthesize the well-known polymer polyacrylamide and to treat effluents from water treatment plants and industrial processes. AA exposure was first linked to neurotoxicity in 1997 in Sweden due to a serious event of environmental pollution by the leakage of a sealant during a tunneling project [1,2,3]. The Swedish National Food Administration and researchers from Stockholm University later announced in 2002 that acrylamide, a toxic and potential carcinogen, is produced in many types of foods prepared or cooked at high temperatures [4]. AA is classified as a Group 2A carcinogen by the International Agency for Research on Cancer [5]. High exposure to AA can affect the nervous system, resulting in muscle weakness, numbness of the extremities, sweating, unsteadiness, etc. [6]. Moreover, animal studies have reported its reproductive toxicity [7]. One of its metabolites, glycidamide (GA), is more cytotoxic than AA [8,9]. The health hazards of AA are well-known in environmental medicine.

Metabolic syndrome encompasses several metabolic abnormalities that include central obesity, insulin resistance, hypertension, dyslipidemia, high triglycerides (TG), and low high-density lipoprotein (HDL) cholesterol and is strongly associated with an increased risk of diabetes, stroke, and cardiovascular disease (CVD) [10]. Metabolic syndrome is primarily characterized by insulin resistance, visceral adiposity, atherogenic dyslipidemia, and endothelial dysfunction [11]. Various diagnostic criteria have been proposed by different organizations over the past decade [12]. The present study adhered to criteria set by the National Institute of Health guidelines as having three or more of the above traits, including those that are controlled by medications [13]. The prevalence of metabolic syndrome increases with age, degree of obesity, and propensity to type 2 diabetes. The exact cause is not known, and multiple factors are known to be involved.

Exposure to hazardous materials can cause various physiological disturbances, resulting in damage to various organ systems, including those involved in metabolism [14]. AA exposure reduces blood insulin levels and causes insulin resistance [15]. Exposure of the general adult population to AA was associated with elevated fasting plasma glucose (FPG) levels, oxidative DNA damage, and lipid peroxidation [16]. Thus, the aim of this study was to evaluate the relationship between exposure to AA and its metabolite GA and metabolic syndrome.

## 2. Participants & Methods

### 2.1. Study Design and Participant Selection

The US National Health and Nutrition Examination Survey (NHANES) is a nationally representative, complex sampling survey that combines interviews and results of physical examinations. All data were acquired from the NHANES website. Use of the NHANES data was approved by the NCHS Research Ethics Review Board, and all participants had provided written informed consent. We included participants aged ≥18 years old in the NHANES dataset from 2003–2006 and excluded persons with missing data. Ultimately, 4813 participants were included. Participants were divided into two groups based on the presence or absence of metabolic syndrome. The clinical definition of metabolic syndrome was provided by the National Cholesterol Education Program’s Adult Treatment Panel III (ATP III) [17] (Table 1).

### 2.2. Measurement of AA and GA

Blood samples were obtained by the laboratory at NHANES, National Center for Health Statistics, Centers for Disease Control and Prevention. The blood specimens were collecting in the of whole blood and separated to different vessels for examination. Specimens for acrylamide- and glycidamide hemoglobin adducts analysis may be fresh or frozen erythrocytes or EDTA-whole blood. The sample solutions used for HbAA and HbGA measurements were subjected to the modified Edman reaction, and the Edman products were isolated and analyzed by high-performance liquid chromatography coupled with tandem mass spectrometry (HPLC/MS/MS). This method is linear for HbAA and HbGA in the range 1.25–80 nmol/L. The bench quality control (QC) pools used in this method comprised three concentration levels (low, medium, and high QC pools), and the results were checked in triplicate after each run [18].

### 2.3. Covariates

Ethnicity was categorized as “Mexican American,” “Other Hispanic,” “non-Hispanic white”, “non-Hispanic black” or “Other Race”. Plasma aspartate aminotransferase (AST), creatinine (Cr), cholesterol, and glucose levels constituted the routine biochemistry profile. Analyses were performed with a Beckman Synchron LX20. Smoking status was determined by participants’ answers to the question “Do you currently smoke cigarettes?” The physical condition interview data provided the health status and medical history as reported by the individual and the agent, including emphysema and angina/angina pectoris.

### 2.4. Statistical Analysis

All data analyses were performed using SPSS (Version 18.0 for Windows, SPSS, Inc., Chicago, IL, USA). We applied the Kolmogorov-Smirnov test for normal distribution. The Chi-square test was used to analyze categorical data, the t-test was used to analyze continuous data. The Mann-Whitney U test was used to analyze HbAA and HbGA concentrations. ANOVA was used to analyze the markers for metabolic syndrome related to HbAA and HbGA concentrations. The extended model was used to adjust for covariates. Values of HbAA and HbGA were log-transformed. Multivariate logistic regression models were used to analyze the effect of exposure to AA and GA on the markers for metabolic syndrome. Multi-variant linear regression analysis was used for HbAA and HbGA concentrations and continuous variables for each marker (waist circumference, blood pressure, and glucose and lipid profiles). Model 1 was unadjusted, whereas Model 2 was adjusted for age, gender, ethnicity, serum AST, serum creatinine, emphysema, angina/angina pectoris, and smoking status. Quartile-based analysis was performed to evaluate the trends in HbAA and HbGA concentrations and the association of prevalence of metabolic syndrome. *p* < 0.05 indicates a statistically significant difference.

## 3. Results

### 3.1. Characteristics of the Study Participants

After screening for eligible participants (*N* = 20470) and accounting for those not attending baseline data collection sessions, 4813 participants were finally available for analysis in this study. The participants were divided into the metabolic syndrome group (*N* = 1064) and the non-metabolic syndrome group (*N* = 3749) (Table 2). The mean age of the non-metabolic syndrome group was lesser than that of the metabolic syndrome group (mean ± SD: 33.97 ± 20.86 vs. 53.54 ± 19.20 years). Higher HbAA and HbGA concentrations were noted in the non-metabolic syndrome group. The mean values of all baseline parameters were higher in the metabolic syndrome group than the non-metabolic syndrome group.

### 3.2. Correlations between the Concentrations of HbAA and HbGA and the Metabolic Syndrome Markers

**Figure 1:**
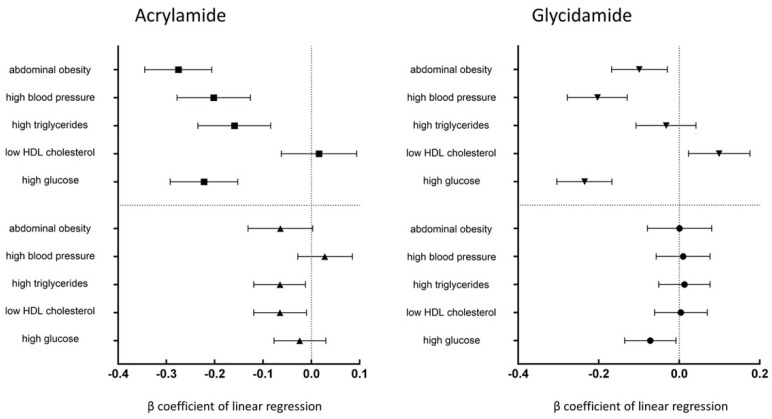
Correlations between concentrations of HbAA and HbGA and markers of metabolic syndrome. The prevalence of high triglycerides, low HDL cholesterol in the adjusted model was inversely associated with the concentrations of HbAA (*p* < 0.05). The prevalence of high FPG in the adjusted model was inversely associated with the concentrations of HbGA (*p* < 0.05).

Table 3 provides HbAA and HbGA concentrations and the number of metabolic syndrome markers. In the adjusted model, log-transformed HbAA concentration inversely correlated with the number of metabolic syndrome markers (3: β coefficient = −0.051; 95% confidence intervals (CI) = −0.141, −0.040; *p* = 0.275 and 4–5: β coefficient = −0.127; 95% CI = −0.229, −0.025; *p* = 0.015). Most results for correlation with log-transformed HbGA were not significant in the adjusted model, except for high glucose level (β coefficient = −0.072; 95% CI = −0.135, −0.008; *p* = 0.027). Increased concentrations of HbAA were noted with high triglycerides and low HDL cholesterol in the adjusted model (*p* < 0.05) (Figure 1). High blood pressure negatively correlated with the concentration of HbAA in Model 1 but was insignificant after multivariate model adjustment (Model 2). In Table 4, the presence of metabolic syndrome was statistically significantly associated with concentrations of HbAA based on quartile analysis in Model 2 (Q2 vs. Q1: OR = 0.604; 95% CI = 0.424, 0.860; Q3 vs. Q1: OR = 0.579; 95% CI = 0.410, 0.817; Q4 vs. Q1: OR = 0.595; 95% CI = 0.404, 0.876; all *p* < 0.05). The association between concentrations of HbGA and metabolic syndrome was not significant.

## 4. Discussion

This is the first study to demonstrate the correlation between metabolic syndrome and HbAA and HbGA levels, which indicate exposure to harmful substances caused by smoking, dietary habits, and occupation. The results revealed a significant negative correlation between metabolic syndrome and HbAA and HbGA concentration. There were no apparent correlations in adjusted model. However, among the metabolic syndrome markers, negative associations with HbAA were observed for high TG and low HDL cholesterol, and negative association with HbGA was observed for high glucose.

The toxicological health hazards of AA exposure are well-known. The main sources of AA in the human body are diet, especially Western-style foods, and occupation. AA has been identified as a carcinogen and is shown to exhibit neurotoxicity, genotoxicity, and deleterious reproductive effects. One of its metabolites, GA, is considered to exhibit even stronger cytotoxicity [7,8]. However, the risk assessment for dietary AA exposure is still under debate. For instance, a negative association of AA exposure with insulin resistance was reported previously [15], and the results from the present study supported this hypothesis.

### 4.1. Acrylamide Exposure and Blood Glucose Regulation

Insulin resistance is central to the development of metabolic syndrome. Increased intracellular fatty acid metabolites contribute to insulin resistance by impairing insulin-signaling pathways. Smoking is a major source of AA exposure, and chronic smoking is associated with high age- and body mass index (BMI)-adjusted plasma insulin levels that are independent of other factors known to influence insulin sensitivity [18]. In a study on female rats [19], exposure to AA significantly increased the FPG level, reduced hepatic glycogen content, and impaired glucose tolerance (i.e., AA exposure significantly promoted gluconeogenesis and glycogenolysis and decreased glycolysis), and damaged islets were also observed. Urinary AA metabolites were positively associated with FPG in a study on the general Asian urban adult population [16], and the results of that study indicated that AA exposure is associated with FPG elevation and increased oxidative DNA damage and lipid peroxidation. In a study on the American adult population [15], AA was found to be associated with reduced serum insulin levels and homeostasis model assessment of insulin resistance. Meanwhile, it has been suggested that the continual intake of trace AA amounts induces characteristically low serum insulin levels in rats [20]. Exposure to endocrine-disrupting chemicals can accelerate T1DM development by several mechanism of action on known type 1 diabetes mellitus triggers, such as immunomodulation, gut microbiota, and vitamin D pathway [21,22,23]. Currently, whether AA exposure could triggered type 1 diabetes mellitus was still lacking of evidence.

### 4.2. Acrylamide Exposure and Lipid Metabolism

Insulin resistance can alter systemic lipid metabolism, which then leads to the development of dyslipidemia and includes high levels of plasma TG and LDL and low levels of HDL cholesterol [24]. Lowering of blood lipid levels, whether by diet or medication, can therefore be regarded as an anti-inflammatory and plaque-stabilizing therapy. The TG fraction and, to a lesser extent, the HDL level can vary considerably depending on the fasting status of the patient [25]. AA exposure promotes adipocyte differentiation and intracellular lipid accumulation in mice, and the effects of AA on mitogen-activated protein kinases (MAPKs) and 5′AMP-activated protein kinase (AMPK)-acetyl-CoA carboxylase activation have also been investigated. Exposure of AA to high-fat diet-fed mice significantly increased their body weight, organ weight, and fat mass [26]. TG molecules represent the major form of storage and transport of fatty acids within cells and in the plasma [27]. AA affects lipoprotein metabolism, which can result in the deterioration of atherosclerosis. Exposure to AA can cause acute inflammatory death, accompanied by hyperlipidemia in zebrafish [28]. The liver is one of the major sites of endogenous cholesterol synthesis. Significant downregulation of genes involved in lipid metabolism could cause lipotoxicity in mice [9]. In the present study, we determined negative correlations between HbAA concentration and high TG and low HDL levels.

### 4.3. Acrylamide Exposure and Bodyweight

Studies on rodents have shown that maternal AA exposure during pregnancy is related to the weight loss of offspring [29]. In subpopulations from the European Prospective Investigation into Cancer and Nutrition (EPIC) study, HbAA was inversely associated with BMI in smokers without adjustment for potential confounders [30]. Cigarette smoking has been associated with increased waist circumference in women younger than 40 years [31]. In a study on chick embryos, low doses of AA significantly increased the total body weight, whereas high doses of AA induced a loss of body weight [32]. Dietary exposure to AA has also been associated with reduced birth weight and head circumference [33].

The mean AA exposure in Europe is estimated to be in the range of 0.31–1.1 µg/kg body weight/day for adults (>18 years old), and the major contributors to the exposure in adults are fried potatoes, coffee, and soft bread, whereas in adolescents and children, the major sources of exposure are fried potatoes, soft bread, potato crisps, and biscuits [34]. The results of a previous study involving the US general population indicated that higher concentrations of HbAA are associated with a decrease in body weight [35]. The results of the present study indicate that HbAA is inversely associated with BMI in the adjusted model.

### 4.4. Acrylamide Exposure and Cardiovascular Disease

AA hemoglobin biomarkers are inversely associated with CVD, including congestive heart failure, coronary heart disease, angina pectoris, heart attacks, and strokes, in people exposed to environmental tobacco smoke and in active smokers. However, there is no significant correlation between hypertension and AA exposure in the risk of CVD [36]. In the mechanism of hypertension, the blood pressure is determined by two components: cardiac output and peripheral resistance. Cardiac output has two determinants: stroke volume and heart rate while peripheral resistance is determined by the vascular structure and function. The neural, endocrine/paracrine, and vascular mechanisms can potentially increase arterial pressure [36,37,38], and the toxic effects of AA on neuron regulation also include sympathetic autonomic neuropathy [6,39]. AA exposure can result in oxidative stress featured by a significant increase in reactive oxygen species (ROS) and malondialdehyde (MDA) levels and consumption of glutathione (GSH) [40]. ROS are involved in inflammation; endothelial dysfunction; cell proliferation, migration, and activation; extracellular matrix deposition; fibrosis; angiogenesis; and vascular remodeling [37]. ROS generation by adipocytes is mediated through the expression and secretion of inflammatory adipokines that impair the regulation of energy homeostasis and vascular function [41,42]. AA exposure disrupts the nitric oxide (NO) signaling pathway, which causes a rostrocaudal difference in the differential expression of nitric oxide synthase (NOS) isoforms nNOS and iNOS in the neocortex and the striatum [43]. In a pediatric chronic kidney disease population, NO-related parameters correlated with blood pressure abnormalities and CVD risk markers, and a positive association between AA metabolites and CVD risk was observed [44]. Although no significant association was found between AA exposure and hypertension, further research is required to assess the potential link between AA exposure and hypertension.

### 4.5. Acrylamide Exposure and Inflammation

A pro-inflammatory state is considered a marker for metabolic syndrome. As mentioned earlier, AA exposure results in oxidative stress featured by increasing ROS and MDA levels and GSH consumption. Moreover, AA activates the nuclear factor erythroid 2-related factor 2 (Nrf2) and nuclear factor-κB (NF-κB) pathways that are regulated by MAPKs [40]. In the activation of the NF-κB pathway, transcription factor Nrf2, a major regulator of antioxidant and cytoprotective genes, is primarily activated in response to oxidative stress. After NF-κB activation, related cytokines, including interleukin (IL)-6, tumor necrosis factor (TNF)-α, granulocyte-colony-stimulating factor (G-CSF), and IL-1β, are released and cell viability decreases [45]. The inflammatory response is determined by concentration-dependent increases in the levels of pro-inflammatory cytokines TNF-α and IL-6, which can cause insulin resistance in adipose tissue, skeletal muscle, and the liver by inhibiting insulin signal transduction [46]. It is logical to assume that AA exposure might induce inflammation because recent studies have demonstrated the cytotoxicity and genotoxicity of AA. Although the relationship between metabolic syndrome and AA exposure was hypothesized as a positive association, the results of this study revealed a negative correlation, as have studies in both animals and humans. Thus, a longitudinal study is required to further determine the outcome of AA exposure and associated health hazards.

### 4.6. Limitations

First, this was a cross-sectional study based on observational and retrospective analyses of existing data from a single period rather than on long-term repeated observations with limited causal inference. Second, the information comes directly from participants’ self-reports, and other data confirming the diagnosis or following dietary habits are limited because AA consumption is one of the main sources of AA exposure. Third, metabolic syndrome is multifactorial, and the incidence of insulin resistance in men and women increases with age and hormone changes, and these variants cannot be adjusted for due to restricted data access. The mean age difference was found in two groups of our study participants. Similar age cohorts studies are required for further evaluation. Fourth, exposure to acrylamide may also be an indicator of exposure to multiple chemicals, including other endocrine-disrupting chemicals, which is hard for covariates adjustment in this study. Fifth, liver genes and enzymes affect the differences in the metabolism of AA exposure in human. Among the components of metabolic syndrome, liver enzymes are also closely related to lipid metabolism and sugar metabolism. In this study, we could not assess the impact of genetic differences between ethnic groups.

## 5. Conclusions

The findings of this study indicate that Hb-adducted AA, the biomarker of exposure to AA is negatively associated with high triglycerides, low HDL cholesterol, markers of metabolic syndrome. Hb-adducted GA, the metabolite of AA in human, is negatively associated with high glucose. As exposure to AA from smoke and food has become a worldwide concern, the findings of this study provide an epidemiological data of AA exposure. The toxic effects of AA include neurotoxicity, reproductive toxicity, genotoxicity, and carcinogenicity. The results of the study indicate that AA could play a role in different stages of progression of metabolic syndrome-related diseases. In the future, the association between long-term exposure and disease progression in different ethnic groups (including dietary habits and smoking status) needs further research to clarify the effects of AA on human health.

## Figures and Tables

**Table 1:** Criteria used for the diagnosis of the metabolic syndrome criteria (NCEP-ATPIII).

Abdominal Obesity	Waist Circumference (Men > 102 cm; Women > 88 cm)
High triglyceride (TG) level ^†^	≥1.69 mmol/L (≥150 mg/dL)
Low HDL cholesterol level	Men: <1.03 mmol/L (<40 mg/dL); Women: <1.29 mmol/L (<50 mg/dL)
High blood pressure ^†^	Systolic ≥ 130 mm hg or diastolic ≥ 85 mm hg
Impaired FPG concentration ^†^	≥5.9 mmol/L (≥100 mg/dL)

For a diagnosis of metabolic syndrome, individuals must meet three or above of the five specific markers. ^†^ If participants were using medications for high triglyceride (TG), hypertension, or diabetes at present, they met the criteria for high TG level, high blood pressure or high fasting plasma glucose (FPG) concentration.

**Table 2:** Characteristics of participants with and without metabolic syndrome.

Variables	*N* = 4813		
	Non-Metabolic Syndrome *N* = 3749	Metabolic Syndrome *N* = 1064	*p* Value
Continuous variables			
Age (years) ^a^	33.97 (20.86)	53.54 (19.20)	<0.001
Acrylamide (pmol/g Hb) ^b^	54.70 (34.70)	49.80 (34.80)	<0.001
Glycidamide (pmol/g Hb) ^b^	51.40 (36.25)	50.00 (36.88)	<0.001
Serum creatinine (μmol/L) ^a^	74.26 (22.98)	82.21 (25.64)	<0.001
Serum AST (U/L) ^a^	24.56 (12.41)	26.28 (11.77)	<0.001
SBP (mmHg) ^a^	116.46 (16.48)	134.36 (21.04)	<0.001
DBP (mmHg) ^a^	64.23 (12.82)	71.64 (15.82)	<0.001
Waist circumference (cm) ^a^	88.84 (15.21)	108.78 (13.66)	<0.001
Serum TG (mmol/L) ^a^	2.36 (1.61)	5.17 (4.32)	<0.001
Serum HDL (mmol/L) ^a^	1.50 (0.39)	1.18 (0.34)	<0.001
FPG (mmol/L) ^a^	5.20 (1.06)	6.69 (2.47)	<0.001
Categorical variables, *n* (%)			
Gender			
Male	2027 (50.5)	573 (49.7)	0.666
Female	1990 (49.5)	579 (50.3)	
Race			
Mexican American	950 (23.6)	277 (24.0)	<0.001
Other Hispanic	123 (3.1)	29 (2.5)	
Non-Hispanic White	1664 (41.4)	622 (54.0)	
Non-Hispanic Black	1091 (27.2)	183 (15.9)	
Other Race—Including Multi-Racial	189 (4.7)	41 (3.6)	
Past history			
Emphysema	52 (2.1)	30 (2.8)	0.257
Angina/angina pectoris	54 (2.2)	57 (5.3)	<0.001
Smoke	483 (41.1)	195 (33.4)	<0.001

^a^ Mean (SD); ^b^ median (IQR); SBP, systolic blood pressure; DBP, diastolic blood pressure; TG, triglycerides; HDL, high-density lipoprotein; AST, aspartate aminotransferase; SD, standard deviation; FPG, fasting plasma glucose.

**Table 3:** Regression coefficients of the presence and number of metabolic syndrome components with log-transformed hemoglobin-adducted acrylamide (HbAA) and hemoglobin-adducted glycidamide (HbGA) concentration.

Variables	Acrylamide (HbAA)				Glycidamide (HbGA)			
	Model 1		Model 2		Model 1		Model 2	
	β (95% CI)	*p* Value	β (95% CI)	*p* Value	β (95% CI)	*p* Value	β (95% CI)	*p* Value
Presence of metabolic syndrome	−0.214 (−0.228, −0.140)	<0.001	−0.031 (−0.089, 0.027)	0.298	−0.119 (−0.193, −0.046)	0.001	−0.001 (−0.070, 0.068)	0.977
Number of metabolic syndrome markers								
1	−0.165 (−0.272, −0.058)	0.002	−0.011 (−0.088, 0.067)	0.789	−0.110 (−0.216, −0.003)	0.044	0.005 (−0.087, 0.097)	0.918
2	−0.301 (−0.405, −0.198)	<0.001	−0.082 (−0.163, −0.002)	0.045	−0.179 (−0.283, −0.075)	0.001	−0.036 (−0.132, 0.060)	0.457
3	−0.319 (−0.429, −0.209)	<0.001	−0.051 (−0.141, −0.040)	0.275	−0.191 (−0.301, −0.081)	0.001	−0.017 (−0.125, 0.091)	0.762
4–5	−0.487 (−0.612, −0.363)	<0.001	−0.127 (−0.229, −0.025)	0.015	−0.278 (−0.402, −0.154)	<0.001	−0.023 (−0.145, 0.098)	0.707
*P* for trend	<0.001		0.013		<0.001		0.570	

Model 1 = unadjusted. Model 2 = Model 1 + age, gender, race-ethnicity, serum AST, serum creatinine, emphysema, angina/angina pectoris, smoking.

**Table 4:** Association of concentrations of HbAA and HbGA and prevalence of metabolic syndrome with quartile analysis.

	HbAA		HbGA	
	OR (95% CI)	*p* Value	OR (95% CI)	*p* Value
Model 1				
Metabolic syndrome	0.997 (0.995, 0.998)	<0.001	0.997 (0.995, 0.999)	0.002
Q2 vs. Q1	0.560 (0.398, 0.788)	0.005	1.002 (0.724, 1.386)	0.992
Q3 vs. Q1	0.559 (0.403, 0.777)	0.002	0.788 (0.577,1.076)	0.134
Q4 vs. Q1	0.462 (0.351, 0.608)	<0.001	0.674 (0.510, 0.890)	0.005
Model 2				
Metabolic syndrome	0.998 (0.996, 1.000)	0.023	1.000 (0.997, 1.002)	0.911
Q2 vs. Q1	0.604 (0.424, 0.860)	0.005	1.070 (0.763, 1.499)	0.696
Q3 vs. Q1	0.579 (0.410, 0.817)	0.002	0.888 (0.637, 1.237)	0.481
Q4 vs. Q1	0.595 (0.404, 0.876)	0.009	1.020 (0.717, 1.452)	0.911

Model 1 = unadjusted. Model 2 = Model 1 + age, gender, race-ethnicity, serum AST, serum creatinine, emphysema, angina/angina pectoris, smoking.

## Data Availability

NHANES data are publicly available at https://wwwn.cdc.gov/nchs/nhanes/Default.aspx (accessed on 27 October 2021).

## Abbreviations

AA, acrylamide; GA, glycidamide; NHANES, National Health and Nutrition Examination Survey; Hb, hemoglobin; FPG, fasting plasma glucose; ALT, alanine aminotransferase; CVD, cardiovascular disease.
